# Mavacamten in Patients With Hypertrophic Cardiomyopathy Referred for Septal Reduction: Week 128 Results From VALOR-HCM

**DOI:** 10.1161/CIRCULATIONAHA.124.072445

**Published:** 2024-11-18

**Authors:** Milind Y. Desai, Kathy Wolski, Anjali Owens, Jeffrey B. Geske, Sara Saberi, Andrew Wang, Mark Sherrid, Paul C. Cremer, Neal K. Lakdawala, Albree Tower-Rader, David Fermin, Srihari S. Naidu, Nicholas G. Smedira, Hartzell Schaff, Zhiqun Gong, Lana Mudarris, Kathy Lampl, Amy J. Sehnert, Steven E. Nissen

**Affiliations:** 1 Hypertrophic Cardiomyopathy Center (M.Y.D., N.G.S.), Cleveland Clinic, OH.; 2 Department of Cardiovascular Medicine, Cleveland Clinic Coordinating Center for Clinical Research (M.Y.D., K.W., P.C.C., S.E.N.), Cleveland Clinic, OH.; 3 Heart Vascular and Thoracic Institute (M.Y.D., K.W., P.C.C., S.E.N.), Cleveland Clinic, OH.; 4 Department of Cardiothoracic Surgery, Heart Vascular and Thoracic Institute (N.G.S.), Cleveland Clinic, OH.; 5 Division of Cardiology, University of Pennsylvania, Philadelphia (A.O.).; 6 Departments of Cardiovascular Diseases (J.B.G.), Mayo Clinic, Rochester, MN.; 7 Cardiovascular Surgery (H.Z.), Mayo Clinic, Rochester, MN.; 8 Department of Internal Medicine, University of Michigan, Ann Arbor (S.S.).; 9 Department of Cardiology, Duke University, Durham, NC (A.W.).; 10 Department of Cardiology, New York University, New York (M.S.).; 11 Division of Cardiology, Brigham and Women’s Hospital, Boston, MA (N.K.L.).; 12 Division of Cardiology, Massachusetts General Hospital, Boston (A.T.R.).; 13 Department of Cardiology, Corewell Health, Grand Rapids, MI (D.F.).; 14 Department of Cardiology, Westchester Medical Center, Valhalla, NY (S.S.N., A.J.S.).; 15 Bristol Myers Squibb, Princeton, NJ (Z.G., L.M., K.L., A.J.S.).

**Keywords:** cardiomyopathy, hypertrophic, mavacamten, septal reduction

## Abstract

**BACKGROUND:**

In severely symptomatic patients with obstructive hypertrophic cardiomyopathy (HCM), VALOR-HCM trial (Study to Evaluate Mavacamten in Adults With Symptomatic Obstructive HCM Who Are Eligible for Septal Reduction Therapy [URL: https://clinicaltrials.gov; Unique identifier: NCT04349072]) reported that mavacamten reduced the short-term need for septal reduction therapy (SRT). The current report examined the longer-term effect of mavacamten through end of treatment at week 128.

**METHODS:**

A double-blind randomized placebo-controlled multicenter trial at 19 sites in the United States included symptomatic obstructive HCM patients referred for SRT (enrollment July 2020 through October 2021). The group initially randomized to mavacamten continued the drug for 128 weeks and the placebo to mavacamten group from week 16 to 128 (112-week exposure). Dose titrations were performed using echocardiographic left ventricular outflow tract gradient and left ventricular ejection fraction measurements. The principal end point was proportion of patients proceeding with SRT or remaining guideline-eligible at week 128.

**RESULTS:**

At week 128, 17 of 108 (15.7%) patients in the total study sample met the composite end point (7 underwent SRT, 1 was SRT-eligible, and 9 SRT-status unevaluable). Additionally, 87 of 108 (80.5%) patients demonstrated ≥1 New York Heart Association class improvement by week 128, and 52 of 108 (48.1%) demonstrated ≥2, with a sustained reduction in resting and Valsalva left ventricular outflow tract gradients of 38.2 mm Hg and 59.4 mm Hg, respectively. Ninety-five of 108 (88%) patients transitioned to commercial mavacamten. Overall, 15 of 108 (13.8%) patients (5.41 per 100 patient-years) had an left ventricular ejection fraction <50% (2 with left ventricular ejection fraction ≤30%; 1 death). Of these, 12 of 15 (80%) continued treatment. New-onset atrial fibrillation occurred in 11 (10.2%) patients (4.55 per 100 patient-years).

**CONCLUSIONS:**

In severely symptomatic obstructive HCM patients, sustained freedom from SRT was observed at 128 weeks, with nearly 90% patients remaining on long-term mavacamten.

**REGISTRATION:**

URL: https://www.clinicaltrials.gov; Unique identifier: NCT04349072.

**Clinical Perspective**

**What Is New?**

- While mavacamten reduces the short−term need for septal reduction therapy (SRT) in patients with obstructive hypertrophic cardiomyopathy, its longer−term impact on the need for SRT remains uncertain.
- At week 128 in VALOR−HCM (Study to Evaluate Mavacamten in Adults With Symptomatic Obstructive HCM Who Are Eligible for Septal Reduction Therapy), 17 of 108 (15.7%) patients met the composite end point (7 underwent SRT, 1 was SRT−eligible, and 9 had unevaluable SRT status); 95 of 108 (88%) chose to transition to commercial mavacamten.
- Overall, 15 of 108 (13.8%) patients (5.41 per 100 patient−years) had a left ventricular ejection fraction <50% (2 with left ventricular ejection fraction ≤30%; 1 death), but 12 of 15 (80%) continued treatment.

**What Are the Clinical Implications?**

- In symptomatic obstructive hypertrophic cardiomyopathy, sufficient and sustained improvement with mavacamten to no longer need SRT, represents a useful noninvasive therapeutic option for SRT−eligible patients.

Hypertrophic cardiomyopathy (HCM), a myocardial disease of hypercontractility and diastolic dysfunction, often leads to worsening dyspnea and reduced exercise capacity due to dynamic left ventricular (LV) outflow tract (LVOT) obstruction and mitral regurgitation. Patients with severe HCM also have an increased risk of heart failure, atrial fibrillation (AF), and sudden arrhythmia-related cardiac death.^1–3^ For the first 60 years, since the original description by Braunwald et al,^4^ standard of care medical therapy aimed at improving patients’ symptoms and quality of life consisted of off-label use of beta blockers, calcium channel blockers, and disopyramide, which had limited efficacy in modifying disease progression or altering the clinical course of HCM. As a result, in patients with symptomatic obstructive HCM who do not respond to traditional medical therapy, septal reduction therapy (SRT) provides excellent symptom improvement, improved quality of life, and long-term survival.^1,3,5–15^ However, optimal results from SRT are only available at specialized centers and due to heterogeneity in outcomes based on procedural volumes, limited number of high-volume SRT centers,^16–19^ and patient preference for better noninvasive alternatives to SRT, there is a need for novel therapies for symptomatic obstructive HCM patients refractory to standard medical therapy.

In previous studies of symptomatic obstructive HCM patients, mavacamten, a selective allosteric and reversible cardiac myosin inhibitor, has demonstrated significant and sustained improvement in LVOT obstruction, symptoms, quality of life, biomarkers, exercise performance and favorable cardiac remodeling.^20–31^ VALOR-HCM (Study to Evaluate Mavacamten in Adults With Symptomatic Obstructive Hypertrophic Cardiomyopathy Who Are Eligible for Septal Reduction Therapy), a phase-3 placebo-controlled trial reported that the addition of mavacamten to maximally-tolerated medical therapy allowed severely symptomatic obstructive HCM patients to experience improved symptom burden, quality of life, mitral regurgitation and biomarkers sufficiently to not need SRT for up to 56 weeks; similar effects were observed in patients who actively crossed over from placebo after 16 weeks.^20,24,25,30^ In addition, previous reports from VALOR-HCM have demonstrated favorable effects on diastolic function and cardiac remodeling, including improvement in LV strain, left atrial (LA) size and function.^23,29,32^ Based on the overall results, mavacamten was approved by the US Food and Drug Administration in 2022 and subsequently in multiple countries around the world. These findings have resulted in updated guidelines that recommend mavacamten as a key therapeutic option in managing symptomatic obstructive HCM patients.^2,3^ However, there is a major unmet need for long-term safety and efficacy data in patients with advanced obstructive HCM. Here, we report the long-term safety and efficacy results from the VALOR-HCM trial through 128 weeks (end of treatment) in patients initially randomized to mavacamten (day 1 to week 128) and patients initially randomized to placebo who transitioned to mavacamten for 112 weeks of exposure (week 16 to week 128).

## Methods

The authors will not make the data, methods used in the analysis and materials used to conduct the research available to any researcher for purposes of reproducing the results or replicating the procedure.

### Study Organization and Oversight

This was a multicenter, randomized, double-blind, placebo-controlled, phase 3 trial conducted at 19 sites in the United States.^33^ The trial was funded by Bristol Myers Squibb (Princeton, NJ) and coordinated by Cleveland Clinic Coordinating Center for Clinical Research and Medpace, a contract research organization (Cincinnati, OH). Details of the academic oversight, study protocol, and statistical analysis plan have been published previously.^33^ The protocol was developed by the sponsor, Cleveland Clinic Coordinating Center for Clinical Research, and Medpace, and approved by Institutional Review Boards at participating centers, with all patients providing written informed consent. An independent Data Monitoring Committee had access to unblinded data during the randomized blinded portion of the trial. After the last patient reached week 128 visit (May 2024), the database was locked, and a complete copy was transferred to Cleveland Clinic Coordinating Center for Clinical Research, where an independent statistician performed statistical analyses.

The first author wrote the manuscript and made final revisions based on comments from other authors, including the sponsor. The authors assume responsibility for the accuracy of the data analyses.

### Study Population

The trial enrolled patients on maximally-tolerated medical therapy, referred for and were considering SRT, based on 2011 American College of Cardiology/American Heart Association (ACC/AHA) guidelines.^34^ Key inclusion criteria were: age ≥18 years, severe dyspnea or chest pain (New York Heart Association [NYHA] class III/IV or class II with exertional syncope or near syncope) despite maximally-tolerated medical therapy (including combination therapy and/or disopyramide). Patients had obstructive HCM (unexplained hypertrophy with a maximum septal wall thickness determined by a core laboratory ≥15 mm or ≥13 mm with family history of HCM) with LVOT gradient at rest or with provocation (Valsalva maneuver or postexercise) ≥50 mm Hg, and a documented LV ejection fraction (LVEF) ≥60%. Patients were referred within the past 12 months for SRT and actively considering the procedure. Patients could elect to proceed to SRT at any time after randomization. Additional inclusion and exclusion criteria have been reported previously and are shown in Table S1.^33^

### Study Procedures

Patients were initially randomized 1:1 to oral mavacamten 5 mg/day or placebo, stratified by SRT type (myectomy or alcohol ablation) and NYHA class. The flowchart of patients is shown in Figure S1. The study protocol and schemata for dose titration, based on LVEF and LVOT gradient have been published previously and shown in the Supplemental Material and Figure S2.^33^ Until week 32, the patients and study staff remained blinded to the original treatment assignment and mavacamten dose; blinded core laboratory LVEF and LVOT gradients were used for drug titration. After 32 weeks, the patients and study staff remained blinded to the original treatment assignment and dose titrations were based on site-based LVEF and LVOT gradients. If the LVEF fell below 50% during treatment, mavacamten was temporarily interrupted with 2- to 4-week follow-up. If LVEF was ≥50% at that time, mavacamten was restarted at 1 dose-level lower. Criteria for permanent discontinuation included 2 LVEF readings <50% at the lowest dose of 2.5 mg or reduction in LVEF to ≤30% at any point during the trial.^20,24,25,33^

### Study End Points

The current study reports 128 weeks of drug exposure (original mavacamten group) or 112 weeks (weeks 16–128) of drug exposure for placebo to mavacamten group. The efficacy end point was the composite of a decision to proceed with SRT or eligibility for SRT according to the 2011 AHA/ACC guidelines.^34^ Patients who discontinued the study or whose response status could not be assessed at week 128 (due to missing data) were classified as SRT-eligible (mavacamten treatment failure). Additional end points included the changes from baseline in postexercise LVOT gradient, NYHA class, Kansas City Cardiomyopathy Questionnaire 23-item Clinical Summary Score, NT-proBNP (N-terminal pro-brain natriuretic peptide), and cTn-I (cardiac troponin I). Echocardiographic end points included changes from baseline in interventricular septal wall thickness, LV mass index, LV systolic and diastolic volume index, left atrial (LA) volume index, and septal E/e. The final study protocol is shown in the Supplemental Material. Safety outcomes included incidence LVEF <50%, death, hospitalization for heart failure, and AF or ventricular tachyarrhythmia.

### Statistical Analysis

The analysis includes all patients initially randomized to mavacamten and placebo patients who transitioned to mavacamten at week 16. Categoric variables are reported as numbers and percentages. The efficacy outcome was the proportion of patients at week 128 meeting SRT eligibility or deciding to proceed with SRT summarized as number and percentage. For placebo patients who transitioned to mavacamten, the week 16 pre-treatment value was used as a baseline for NHYA class assessment, laboratory, and echocardiographic measurements. At baseline, continuous variables are presented as mean (SD) if normally distributed or median (Q1–Q3) if not normally distributed. Changes from baseline for echocardiographic variables are summarized using mean and 95% CI, and changes from baseline in NT-proBNP and cTn-I are reported as median and approximate distribution-free 95% CIs. Analysis was performed using SAS version 9.4 (SAS Institute, Inc., Cary, NC).

## Results

### Study Population

Of 112 highly symptomatic obstructive HCM patients,^20^ 108 qualified for week 128 evaluation, as described previously. All patients initially randomized to mavacamten (n=56), and those who transitioned from placebo to mavacamten after 16 weeks (n=52) were included.

The 4 patients excluded from the original placebo group included 2 patients who underwent SRT during the first 16 weeks and 2 who discontinued the study before 128 weeks.^20^ Of the remaining patients, the mean age was 60.3 years, and 50% were males. All patients were symptomatic (94% NYHA class III/IV) on maximally tolerated HCM therapy with most on beta-blocker monotherapy (46%). The mean±SD LVEF was 68±4% and peak resting, Valsalva, and postexercise LVOT gradients were 50±31, 77±30, and 84±35 mm Hg, respectively. Baseline characteristics are shown in Table 1.

**Table 1.**
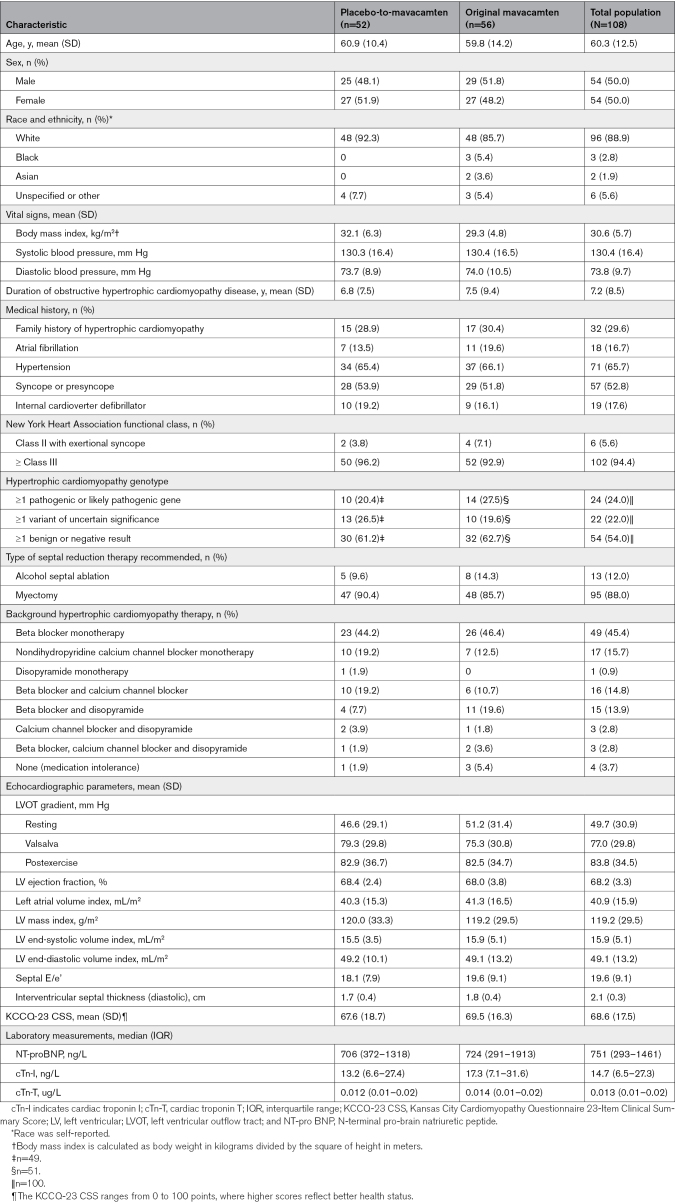
Patient Characteristics of the Trial Population

### Study End Points

**Figure 1.**
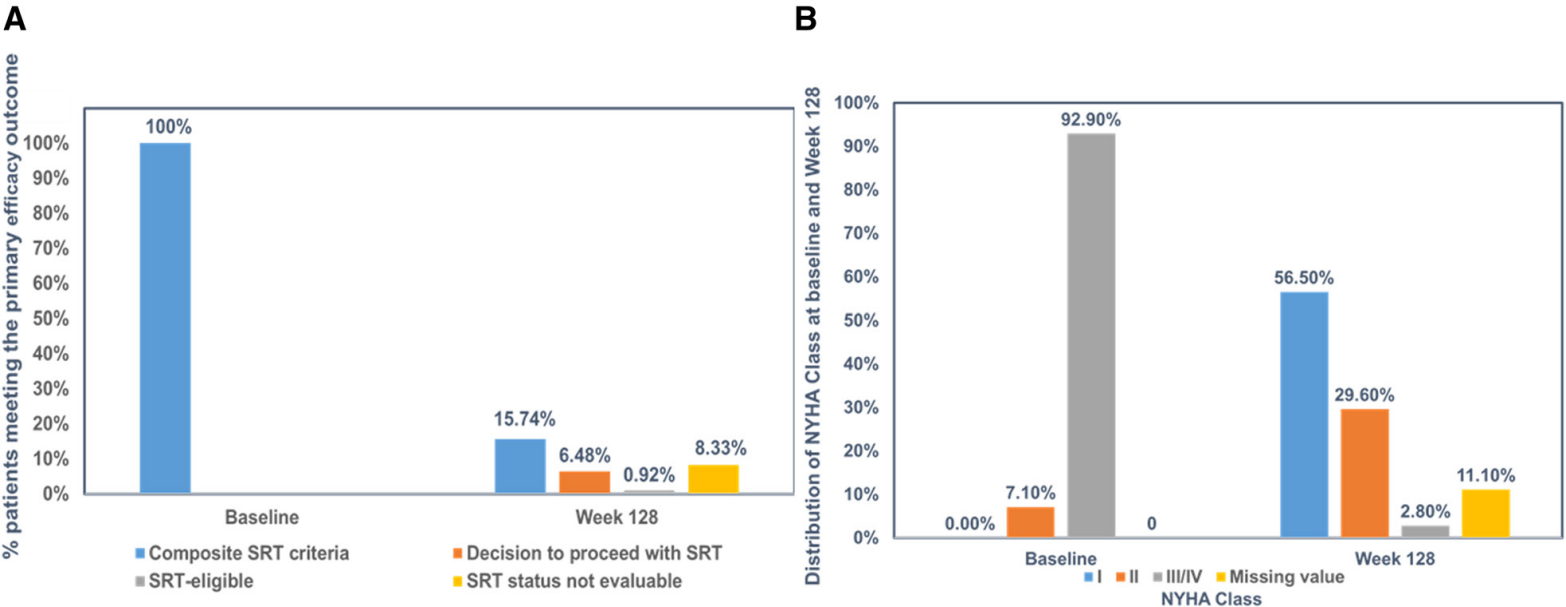
**Primary and key efficacy parameters from baseline to week 128 in VALOR-HCM trial. A**, Primary composite end point from baseline to week 128. **B**, Improvement in New York Heart Association (NYHA) class from baseline to week 128. SRT indicates septal reduction therapy; and VALOR-HCM, Study to Evaluate Mavacamten in Adults With Symptomatic Obstructive HCM Who Are Eligible for Septal Reduction Therapy.

At week 128, 17 of 108 (15.7%) patients in the total study sample met the composite end point (7 underwent SRT, 1 SRT-eligible and 9 SRT-status unevaluable; Figure 1). Patients were classified as unevaluable if they could not be assessed for the composite end point at week 128. Seven of the 9 patients exited the study before week 128 for various reasons (1 lost-to-follow up, 1 death, 2 withdrew consent, 2 met criteria for permanent discontinuation of mavacamten, and 1 was withdrawn because of severe noncompliance). An additional 2 patients completed week 128 follow-up but did not have sufficient information (NYHA class and LVOT gradient assessment) to determine SRT eligibility guideline criteria. Only 8 of 56 (14.3%) in the original mavacamten group and 9 of 52 (17.3%) in the placebo to mavacamten group continued to meet the composite efficacy end point (Table 2). Of these, only 7 of 108 (6.4%) patients (3 of 56 [5.4%] from the original mavacamten group and 4 of 52 [7.7%] from the placebo to mavacamten group) chose to undergo SRT. Details of patients meeting the composite end point, but not undergoing SRT are shown in Table S2. A total of 7 (6.5%) patients discontinued the study before the final visit (3 withdrew consent, 1 was lost to follow-up, 1 died, 1 found ineligible postenrollment, and 1 was removed because of noncompliance). At end of treatment, 95 patients chose to transition to commercial mavacamten, while one is currently awaiting commercial insurance authorization. The data on patients who chose to undergo SRT at week 56, along with their postoperative outcomes, have been published previously.^25^ Between weeks 56 and 128, only 1 additional patient (in the original placebo group) underwent SRT.

**Table 2.**
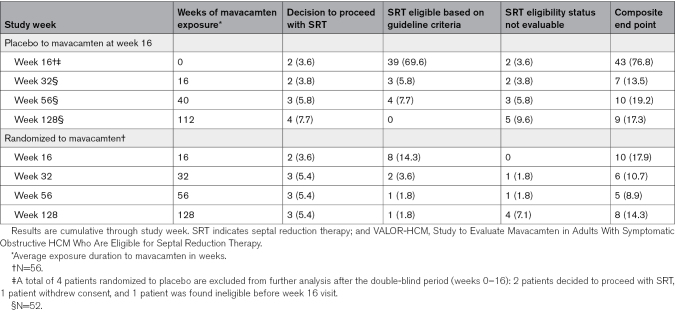
Primary Efficacy Outcome in the VALOR-HCM Trial

**Figure 2.**
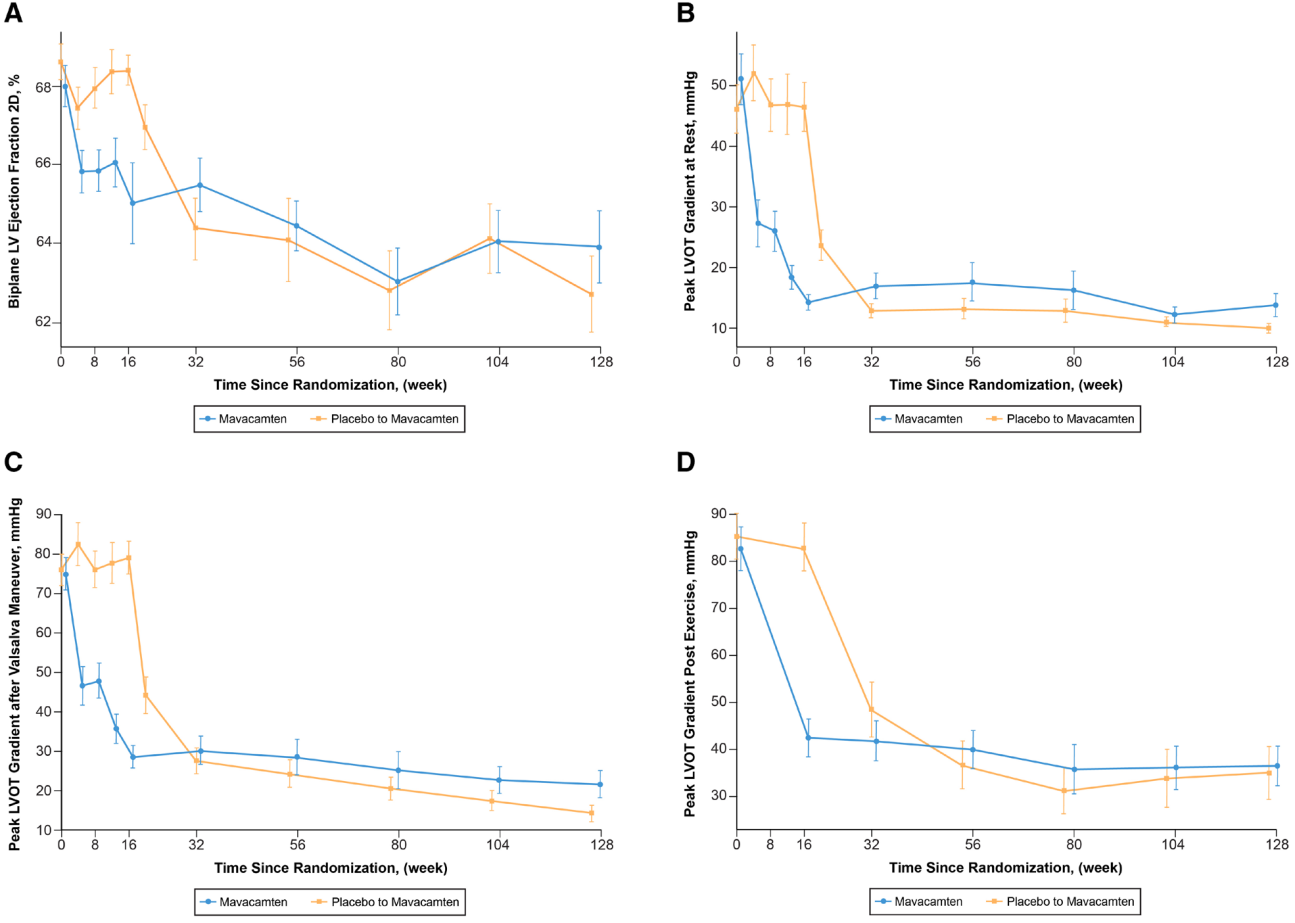
**Key echocardiographic efficacy and safety parameters from baseline to week 128 for the original mavacamten and the placebo to mavacamten groups. A**, Change in left ventricular (LV) ejection fraction. **B**, Improvement in peak left ventricular outflow tract (LVOT) gradient at rest. **C**, Improvement in peak left ventricular outflow tract gradient after Valsalva maneuver. **D**, Improvement in peak left ventricular outflow tract gradient after stress echocardiography. Plotted values in **B** through **D** represent means and standard errors.

In the total study sample, 87 of 108 (80.5%) patients demonstrated ≥ 1 and 52 of 108 (48.1%) demonstrated ≥ 2 NYHA class improvement by week 128 (Figure 1). Within the original mavacamten group, 48 of 56 (85.7%) demonstrated ≥1 and 30 of 56 (53.6%) demonstrated ≥2 NYHA class improvement by week 128. In the placebo-to-mavacamten group, 39 of 52 (75.0%) patients had ≥1 and 22 of 52 (42.3%) ≥2 NYHA class improvement at week 128 (Table 3). Severe systolic anterior motion of the mitral valve was present in 50 of 92 (54.3%) and 3 of 92 (3.3%) patients at baseline and week 128, respectively. Similarly, 22 of 90 (24.4%) and 10 of 90 (11.1%) patients, had at least moderate mitral regurgitation at baseline and week 128, respectively. Data on additional functional, laboratory and hemodynamic parameter improvements from baseline (in the 2 subgroups, as well as the total study sample) are shown in Table 3 and Figures 2 through 4.

**Table 3.**
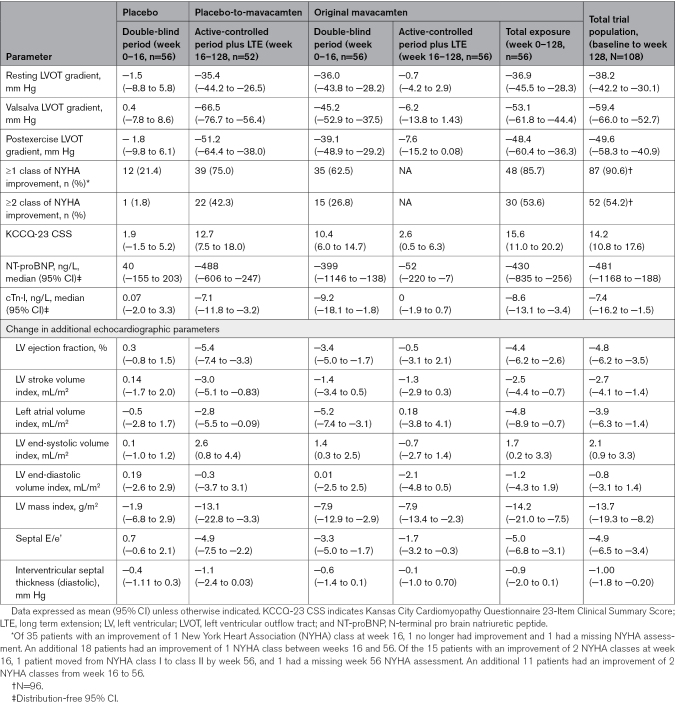
Changes in Functional, Laboratory, and Echocardiographic Parameters

**Figure 3.**
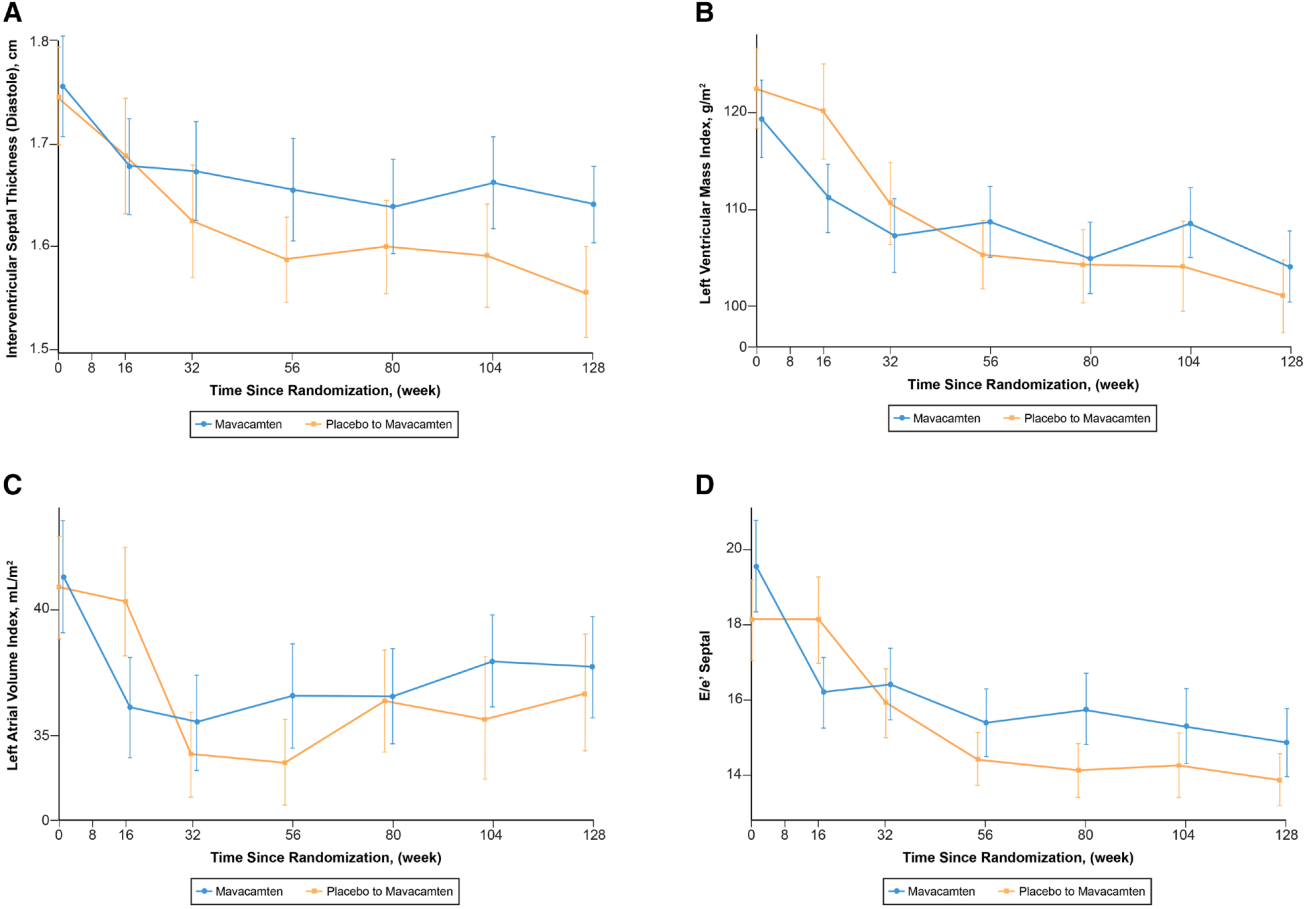
**Additional echocardiographic parameters from baseline to week 128 demonstrating favorable cardiac remodeling for the original mavacamten and the placebo to mavacamten groups. A**, Improvement interventricular wall thickness. **B**, Improvement in left ventricular mass index. **C**, Improvement in left atrial volume index. **D**, Improvement in E/e’. Plotted values in **B** through **D** represent means and standard errors.

**Figure 4.**
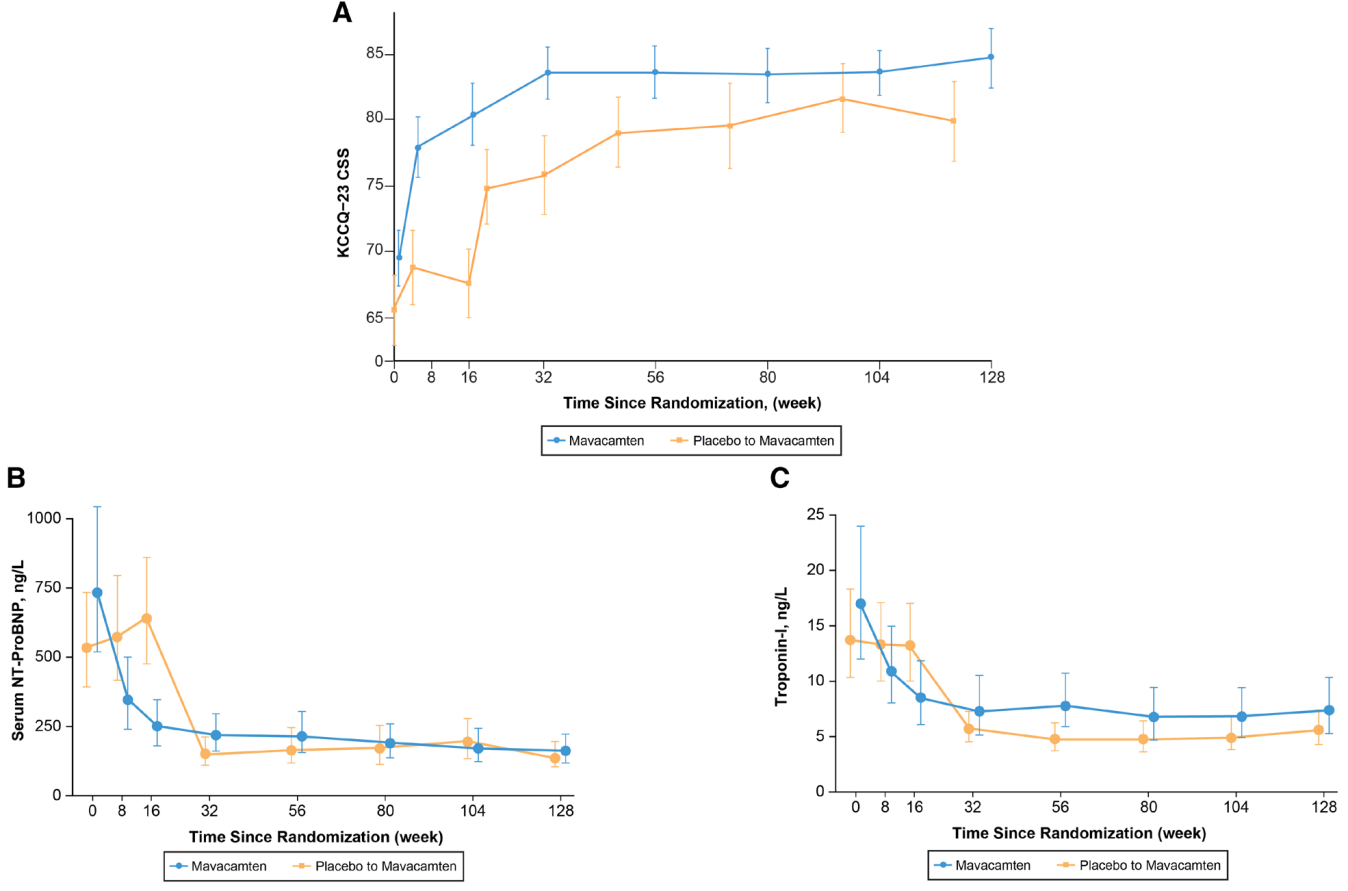
**Changes in biomarkers and health status from baseline to week 128 demonstrating favorable cardiac remodeling for the original mavacamten and the placebo to mavacamten. A**, Improvement in Kansas City Cardiomyopathy Questionnaire 23-Item Clinical Summary Score (KCCQ-23 CSS). **B**, Improvement NT-pro-BNP (N-terminal pro brain natriuretic peptide). **C**, Improvement in cTn-I (troponin I).

The distribution of the final mavacamten doses at week 128 for the entire study sample were as follows: 2.5 mg (n=14 [13%]), 5 mg (n=32 [30%]), 10 mg (n=34 [31%]), and 15 mg (n=28 [26%]), respectively. Between weeks 56 and 128, 25 patients had mavacamten dose titration (up-titration of dose in 20 patients [for persistently elevated LVOT gradient and symptoms] and down-titration in 5 [due to LVEF reduction to <50%]). The data (available in 93 patients) on adjustment of standard background HCM therapies is shown in Table S3. At week 128, 7 (8%) patients were on no background HCM therapy (vs 4 [4%] at baseline), 72 (77.4%) on monotherapy (vs 58 [62.4%] at baseline), 14 (15.1%) on dual therapy (vs 29 [31.1%] at baseline) and none on triple therapy (vs 2 [2.2%] at baseline).

### Safety

Aggregate safety outcomes and adverse events are reported in Table 4 as exposure-adjusted incidence per 100 patient-years (total duration of the trial as well as between weeks 56 and 128). In addition to COVID-19 infection (occurring in 41 [37.9%] patients), the most common relevant adverse events were fatigue and dizziness, occurring in 18 (16.7%) and 16 (14.8%) patients, respectively. From day 1 through week 128, LVEF reduction to < 50% and new-onset AF were observed in 15 (13.9%) and 11 (10.2%) patients, respectively (for details, see Table S4). Of note, 18 events of LVEF <50% occurred in 15 unique patients. Of these, 5 patients had an LVEF reduction to < 50% and 7 patients had new AF between weeks 56 and 128. Of those 5 patients, 3 were new patients who experienced LVEF < 50% for the first time, and 2 had a second LVEF reduction to <50%. This translated into 5.41% and 4.55% exposure-adjusted incidence per 100 patient-years, respectively. Mean LVEF remained within normal limits in both groups (Figure 2A). As reported previously, between Day 1 and Week 56, 3 patients had permanent discontinuation of mavacamten per protocol criteria: 2 for LVEF < 30% and 1 patient for LVEF < 50% on the lowest dose of 2.5 mg on 2 different study visit echocardiograms through week 56. The second occurrence was at week 56. No additional patients required permanent discontinuation between weeks 56 and 128. The remaining 12 patients (11.1%) required mavacamten treatment interruption for LVEF < 50%. Per protocol, all were restarted on a lower dose (10, 5, or 2.5 mg, dependent on previous dose) after a 2 to 4-week pause. All 12 patients had LVEF improvement to ≥ 50% in follow-up and continued treatment.

**Table 4.**
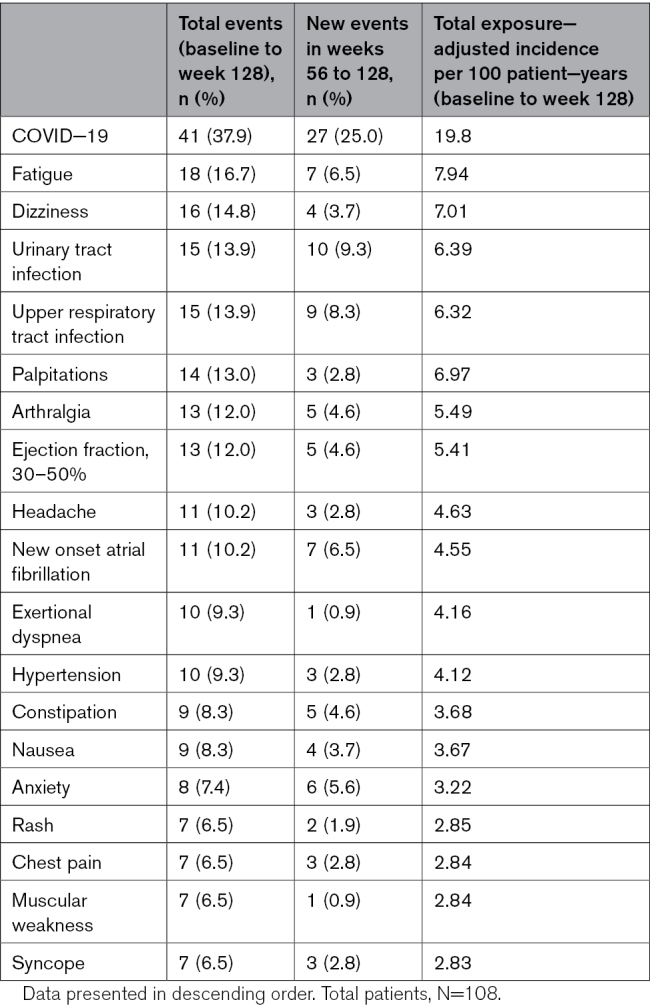
Incidence of Exposure−Adjusted Treatment Emergent Adverse Events per 100 Patient−Years

**Table 5.**
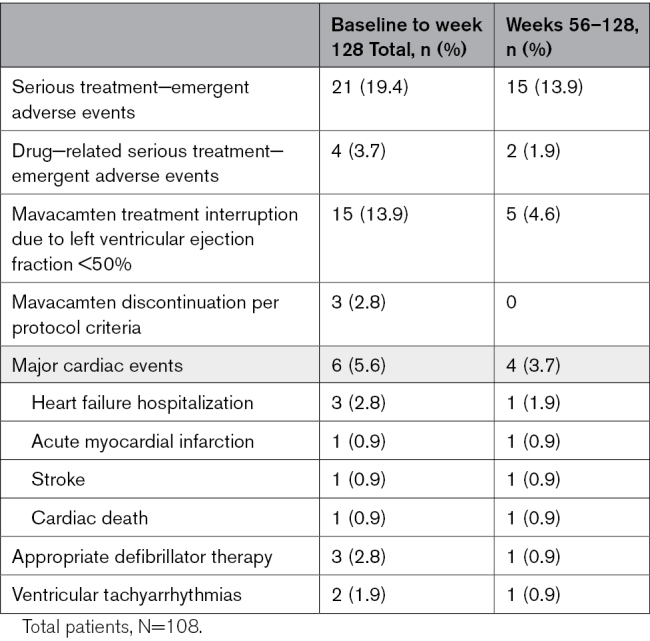
Summary of Patients With Treatment−Emergent Adverse Events, Drug Interruptions and Discontinuations

## Discussion

The current end of treatment report from the VALOR-HCM trial details the longer-term effects of mavacamten through week 128 in patients with symptomatic, obstructive HCM who were eligible and referred for SRT. The main finding of this study is that mavacamten treatment resulted in a low percentage of patients who were eventually treated with SRT or remained guideline-eligible for SRT. This suggests that mavacamten is an efficacious medical therapy in the long-term as an alternative to SRT. Indeed, at the end of the trial, the majority of patients chose to transition to commercial mavacamten.

Patients treated with mavacamten demonstrated a significant and sustained improvement in symptoms and quality of life in the longer-term. At week 128, 80.5% and 48.1% patients demonstrated ≥1 and ≥2 NYHA class improvement from baseline, respectively. In addition, there was significant and sustained improvement in Kansas City Cardiomyopathy Questionnaire 23-item Clinical Summary Score (14.2-point change), which was much higher than what was previously reported in various other heart failure trials.^28^ The current data adds to a recent report from the VALOR-HCM trial, which provided a detailed analysis of significant and sustained health status improvement assessed by various Kansas City Cardiomyopathy Questionnaire domains up to week 56.^30^

In addition to previously described shorter-term improvements, the prolonged exposure to mavacamten demonstrated sustained and favorable changes noted on serial echocardiography in the entire study sample.^20,23–25,32^ This was manifested as a significant and sustained improvement in various echocardiographic parameters, including resting and provoked LVOT gradients, biomarkers, E/e’, interventricular septal thickness, LV mass index, and LA volume index at week 128. Additionally, mavacamten has also been shown to have a significant and sustained favorable improvement in LV global longitudinal strain and LA strain up to week 56.^29,31^ These findings suggest favorable longer-term consequences of reduced LVOT gradient, along with favorable lusitropic effects of mavacamten.^21,29^

Because of the mechanism of action of cardiac myosin inhibitors, avoiding excessive reduction in LV systolic function requires careful monitoring for patient safety. VALOR-HCM wholly used echocardiography for dose titration and safety assessment, a more practical, clinically relevant, and widely available approach compared with assessment of plasma mavacamten concentration.^33^ The echocardiographic measurements were blinded and adjudicated by the core-lab until week 32 and subsequently were site-read. This enabled us to test the feasibility of dual strategies of (1) a blinded core laboratory echocardiographic assessment (reducing bias) during the initial randomized phase, and (2) the safety and practicality of a site-based echocardiographic assessment during the long-term open-label extension phase of the trial. Indeed, we previously demonstrated a high correlation of LVEF and LVOT gradients, as reported by individual sites versus the core laboratory.^25^ It is important to recognize that the patients and investigators remained blinded to the actual drug dosage throughout the study.

While 12 mavacamten-treated patients temporarily discontinued therapy for transient reduction in LVEF during the trial, all remained asymptomatic and resumed treatment at a lower dose. However, as described previously,^25^ 3 met the criteria for permanent drug discontinuation after initiation of mavacamten treatment, 1 of whom experienced sudden cardiac death. The increased rate of LVEF reduction, compared with previous reports,^21,35^ is likely related to: (1) aggressive and blinded dose-titration in the current study in which 26% of patients were on the highest dose of mavacamten (15 mg); and (2) more symptomatic patients with advanced disease requiring higher doses. Similar-sized cohorts of patients being treated with commercial mavacamten show lower proportions of highly symptomatic patients (NYHA class III) and fewer treated with the highest dose of mavacamten (15 mg).^36,37^ This is likely reflected in a much lower number of patients requiring temporary discontinuation of mavacamten (range, 2–3%) in real-world experiences. In addition, the proportion of patients with heart failure hospitalizations, permanent mavacamten discontinuation or downstream SRT was also extremely small in these nontrial cohorts. These findings may suggest a sample bias within the clinical trial populations, tending to attract more symptomatic patients with more advanced disease than the overall population of patients with obstructive HCM. Future longer-term studies may help determine the long-term safety of prolonged administration of mavacamten for sustained avoidance of SRT.

Another safety end point of interest is the incidence of new onset AF while on mavacamten therapy, which was at 4.92 per 100 patient exposure–years. In shorter term, placebo-controlled studies, the incidence of AF has been similar for patients treated with mavacamten versus placebo.^21^ Another recent long-term study of mavacamten treatment for 252 weeks reported similar incidence of new AF events (4.5 per 100 patient-years).^35^ For comparison, the incidence of AF after SRT has been reported to be 34% overall and 23% de novo AF after myectomy, with 19% developing AF >30 days after their surgery.^38^ In a disease with a relatively high prevalence of AF, it would be extremely difficult to convincingly demonstrate a causal relationship between these therapies and AF. However, in a detailed analysis from the VALOR-HCM trial, we have demonstrated that patients with a history of AF had higher LA volume index and significantly lower LA strain parameters versus those without a history of AF. While the findings of LA volume index regression and improvement in LA strain while on longer-term mavacamten are reassuring, the subgroup with a previous history of AF did not demonstrate an improvement in LA strain parameters at week 56.^31^ Whether they translate to reduced incidence of AF in such patients remains to be proven. In an additional report, as described previously, prior history of AF was higher in patients requiring mavacamten interruption versus remainder of the VALOR-HCM study sample, and on exploratory analysis, a prior history of AF was significantly associated with likelihood of developing LVEF <50%.^29^

Current background HCM therapies, such as beta blockers, nondihydropyridine calcium channel blockers, or disopyramide, while modestly improving symptoms in some symptomatic obstructive HCM patients, have significant side effects resulting in intolerance and noncompliance. In the current report, 37 of 108 (34%) of participants were on at least 2 standard-of-care therapies at baseline. We demonstrate that only 14 of 108 (13%) required 2 or more therapies in addition to mavacamten. We demonstrate that in ≈25% of patients, we were able to streamline patients to a single background HCM therapy (the most common was beta blockers) and reduce the use of 2 or 3 background HCM therapies by >50%. It is likely that some of these adjustments were attributable to factors other than initiation of mavacamten (eg. weight loss, other unwanted side effects, among others). However, in real-world practice, we have also demonstrated a reduction in doses of background HCM therapies.^36^

Increasing use of mavacamten has brought the discussion about the role of SRT into focus. While SRT, especially if performed at a high-volume center, remains a class I recommendation for symptomatic obstructive HCM patients, there is substantial heterogeneity in SRT outcomes with a high risk of morbidity and mortality in low-volume centers.^16–19^ A recent analysis demonstrated that despite a significant inverse relationship between SRT volumes and longer-term outcomes; 70% of SRTs were performed in low-volume centers in the United States.^39^ Beause there are only a few high-volume SRT centers, patient access to optimal SRT results is limited. There is an unmet need for better noninvasive alternatives to SRT for highly symptomatic obstructive HCM patients.^16,40^ Continued shared decision-making dialogue between patients and healthcare providers will remain important in the future, especially with more mainstream use of this drug after regulatory approval. Future cost–benefit analyses will also help guide treatment algorithms.

The current data on mavacamten from VALOR-HCM must also be put in the context of aficamten, the second cardiac myosin inhibitor currently under investigation. SEQUOIA-HCM (Study to Evaluate the Efficacy and Safety of Aficamten Compared to Placebo in Adults With Symptomatic Obstructive HCM), a phase III trial recently reported positive results.^41^ While the drugs have not been directly compared to each other, there were key differences in overall trial designs,^42^ including: (1) differences in inclusion criteria based on NYHA class (VALOR-HCM required NYHA classes III–IV or NYHA class II with exertional syncope or near syncope; SEQUOIA-HCM included those who were in NYHA class II or III); (2) differences in primary end points of SRT eligibility in VALOR-HCM versus change in peak oxygen consumption in SEQUOIA-HCM; (3) a significantly greater proportion of patients on background HCM therapy in VALOR-HCM versus SEQUOIA-HCM. Despite these differences, similar short-term improvements in LVEF, LVOT gradients, and NYHA class were achieved in both trials. In addition, while the present study demonstrated that the majority of patients did not need, or were no longer eligible for, SRT during follow-up, in a subgroup of SEQUOIA-HCM, duration of SRT eligibility was significantly reduced. Whether there are differences in long-term efficacy of these 2 drugs remains to be studied.

### Limitations

The efficacy end point was driven by a reduction in guideline eligibility for SRT rather than the decision of patients not to proceed with SRT. Whether mavacamten can prevent other adverse outcomes favorably affected by SRT such as sudden death, cannot be assessed with the limited sample size and duration of this trial. The current study included predominantly White patients treated at high-volume HCM centers in the United States with established good outcomes for SRT procedures; and further extrapolation in different ethnicities and regions of the world may be difficult.

## Conclusions

In highly symptomatic obstructive HCM, mavacamten treatment in patients taking maximally tolerated medical therapy reduced the long-term need for SRT with persistent improvements in LVOT gradients, symptoms, and quality of life. There was favorable cardiac remodeling, and the treatment was well tolerated. Because of the limited availability of high-volume SRT centers, sufficient improvement with medical therapy so that patients no longer need SRT represents a useful therapeutic option.

## Article Information

### Acknowledgments

Bristol Myers Squibb policy on data sharing may be found at https://www.bms.com/researchers-and-partners/independent-research/data-sharing-request-process.html.

### Sources of Funding

VALOR-HCM (Study to Evaluate Mavacamten in Adults With Symptomatic Obstructive HCM Who Are Eligible for Septal Reduction Therapy) was funded by Bristol Myers Squibb (Princeton, NJ).

### Disclosures

M. Desai reports consulting for Bristol Myers Squibb, Cytokinetics, Tenaya, and Medtronic; and research support to Cleveland Clinic from Bristol Myers Squibb, Cytokinetics, and Tenaya. A. Owens reports consulting for Bristol Myers Squibb, Cytokinetics, Pfizer, Biomarin, Tenaya, Lexicon, Stealth, Edgewise, and Renovacor; and receives grant support for research from Bristol Myers Squibb. Dr Saberi reports consulting for Bristol Myers Squibb and Cytokinetics. Dr Lakdawala has received consulting incomes from Bristol Myers Squibb, Pfizer, Tenaya, Cytokinetics and Akros and research support from Bristol Myers Squibb and Pfizer. Drs Naidu, Wang, Sherrid, and Tower-Rader report consulting for Bristol Myers Squibb and Cytokinetics. Drs Smedira and Geske report consultation with Bristol Myers Squibb. Dr Fermin reports conflicts from Bristol Myers Squibb (consulting, speaking), Pfizer (consulting), BridgeBio (consulting, speaking). Drs Nissen and Wolski work for C5 Research and are employees of Cleveland Clinic, which received payments for the present research from Bristol Myers Squibb. Drs Gong, Mudarris, Sehnert, and Lampl are employees of Bristol Myers Squibb. Dr Cremer was employed during the conduct of the trial by Cleveland Clinic, which received payments for current research from Bristol Myers Squibb.

The other authors report no conflicts.

### Supplemental Material

Tables S1–S4

Figures S1 and S2

Clinical Study Protocol
